# Human Transporter Database: Comprehensive Knowledge and Discovery Tools in the Human Transporter Genes

**DOI:** 10.1371/journal.pone.0088883

**Published:** 2014-02-18

**Authors:** Adam Y. Ye, Qing-Rong Liu, Chuan-Yun Li, Min Zhao, Hong Qu

**Affiliations:** 1 Center for Bioinformatics, State Key Laboratory of Protein and Plant Gene Research, College of Life Sciences, Peking University, Beijing, China; 2 Behavioral Neuroscience Research Branch, NIH-IRP (NIDA), Baltimore, Maryland, United States of America; 3 Institute of Molecular Medicine, Peking University, Beijing, China; 4 Peking-Tsinghua Center for Life Sciences, College of Life Sciences, Peking University, Beijing, China; Huazhong University of Science and Technology, China

## Abstract

Transporters are essential in homeostatic exchange of endogenous and exogenous substances at the systematic, organic, cellular, and subcellular levels. Gene mutations of transporters are often related to pharmacogenetics traits. Recent developments in high throughput technologies on genomics, transcriptomics and proteomics allow in depth studies of transporter genes in normal cellular processes and diverse disease conditions. The flood of high throughput data have resulted in urgent need for an updated knowledgebase with curated, organized, and annotated human transporters in an easily accessible way. Using a pipeline with the combination of automated keywords query, sequence similarity search and manual curation on transporters, we collected 1,555 human non-redundant transporter genes to develop the Human Transporter Database (HTD) (http://htd.cbi.pku.edu.cn). Based on the extensive annotations, global properties of the transporter genes were illustrated, such as expression patterns and polymorphisms in relationships with their ligands. We noted that the human transporters were enriched in many fundamental biological processes such as oxidative phosphorylation and cardiac muscle contraction, and significantly associated with Mendelian and complex diseases such as epilepsy and sudden infant death syndrome. Overall, HTD provides a well-organized interface to facilitate research communities to search detailed molecular and genetic information of transporters for development of personalized medicine.

## Introduction

Transport proteins (transporters) are membrane channels and molecular pumps to facilitate exchange of ions, small molecules, macromolecules, and drugs across membranes [1]. The movement of biochemical compound through membrane is critical to absorption, distribution, metabolism, and excretion (ADME) of nutrients, neurotransmitters, and drugs [2]–[5], [6]. The dynamic partnerships of transporter with other signaling molecules in subcellular locations are regarded as essential processes for cellular function. Attenuation of transporter gene functions by polymorphisms often contributes to complex human diseases and individual drug responses [4], [5]–[9]. How do transporters cooperate with intracellular signaling systems and metabolic systems to give precise control of transmembrane trafficking? Although crystal structures have shed light on the regulatory mechanisms of a few individual transporters as gateway for metabolites and signals in the past decade [10], the global features of transporter genes are still not clear. Recent advances in high throughput technologies, such as mass spectrometry (MS), genome-wide association study (GWAS), and next-generation sequencing (NGS), provide abundant complementary data to study transporting processes or the effects of transporters on normal cellular processes and various disease states [11], [12]. A comprehensive database of human transporters is required to incorporate the most updated high throughput data in an intuitive search engine.

There are two types of previous transporter databases: general transporter collections and gene family specific collections. The earlier general transporter databases include TCDB (Transporter Classification Database), TransportDB, KEGG (Kyoto Encyclopedia of Genes and Genomes), HMTD (Human Membrane Transporter Database), and TSdb (Transporter substrate database). TCDB is dedicated to transporter classification based on functional and phylogenetic information [13], which contains 513 human, 364 mouse, and 165 rat transporters. TransportDB focuses on prediction cytoplasmic membrane transporters for comparative studies with 1,022 human and 1,090 mouse transporters [14]. In KEGG PATHWAY and BRITE database, there are 870 transporter orthology groups in prokaryotes and eukaryotes, which maps to 420 human genes [15]. HMTD (Human Membrane Transporter Database) is specific for drug transport studies and pharmacogenomics with 287 human transporters [16]. TSdb (Transporter substrate database) is constructed to annotate substrates of transporters [17]. Another type of gene family specific transporter databases only focus on specific transporter families including ABCdb (ATPbinding cassette transporters database) [18], MTDB (Medicago truncatula transporter database) [19], and SLCdb (Caenorhabditis elegans SLC homologue database, http://www.wormslc.org/). However, most of the transporter databases were derived from low throughput data, and without integrating high throughput expression and polymorphism data, or without systematically updating for recent pharmacogenetic data.

A lack of integration of these high throughput data across functional, pharmaceutical, and genetic studies hampers our understanding of the molecular mechanisms of transporter related diseases. Some transporters can influence drug efficacy, and their activity can also be affected by some drugs, thus when two or more drugs are coadministered, their dosage may need adjustment [20]. In addition, natural variants such as single-nucleotide polymorphism (SNP) may also affect transporter activity [21], and may sometimes make the protein more sensitive to drug [22]. Data integration will be useful for generating new hypothesis, such as dosage and safety warnings on drug coadministration or population polymorphism, refining our understanding of cellular transporting system in human disease states and development of transporter gene based pharmacogenetics [23]. To provide insight into human transporter systems, we collected 1,555 human non-redundant transporter genes and constructed Human Transporter Database (HTD), a repository for dynamic storage of the ever-increasing bioinformatics on transporter genes in light of personalized medicine.

We extensively annotated human transporter genes (HTGs) from the perspective of sequences, functions, drugs, diseases, pharmacogenetics, genetic variations, interactions, and gene expressions. We noted that the human transporters were enriched in fundamental biological processes and involved in a number of complex human diseases. Overall, HTD provides a publicly accessible resource and a searchable database for communities to explore the human transporters gene families, functional substrates, expressions and polymorphisms in a global way. It is freely available at http://htd.cbi.pku.edu.cn.

## Materials and Methods

### Collection of Human Transporter Genes

We specifically defined transporters as the membrane proteins facilitating materials (mostly molecules or ions) transporting across membrane. In order to get precise descriptive keywords for transporters, we extensively reviewed 1,178 human transporter genes integrated from NCBI Gene database and four relevant transporter datasets: (i) Transporter classification (TC) systems; (ii) TransportDB; (iii) Transporter family and gene list from HMTD; and (iv) KEGG BRITE transporter (ko02000) and solute carrier family (ko02001). Based on the transporter definition, gene description, and GO annotation in NCBI Gene database, we compiled 54 keywords precisely related to transporter gene names and functions (Table S1). In this process, we excluded some keywords irrelevant with membrane transporting such as “fatty acid binding”, which mainly represented apolipoproteins, the proteins bind lipids and transport lipid through circulatory system, and are seldom embedded in cellular membranes for transporting functions.

Using the 54 keywords, we utilized NCBI E-search interface to implement complex query against NCBI Gene database. In total, 1,592 human genes were obtained. In this process, pseudogenes were included, as they may play regulatory roles on transporter related biological processes [24]. Based on gene description, alias, GO annotation, and domain feature, we manually removed those genes irrelevant with transporter function. Further we performed BLAST similarity alignment with these refined genes against all protein sequences in the human genome to include less annotated genes but with high sequence similarities with curated transporter genes. Through additional manually checking, 1,555 human transporter genes with high confidence were stored in our HTD database.

### Gene Annotation

To systematically mine the biological mechanism related to transporter genes, we annotated all transporters in our HTD with extensive functional information. The statistics of those annotation entries in HTD was listed in Table S2. We first extracted basic information including gene symbol, annotation, and function from NCBI Gene database [25], GO annotation, protein sequence and features from UniProt [26], protein domain annotation from InterPro [27], Homolog GeneID mapping data from NCBI HomoloGene [28], SNPs with minor allele frequency or population genetic information from dbSNP [29] and HapMap [30], CNV data from DGV (31), protein-protein interaction information from HPRD [32], expression data from NCBI UniGene [25], Allen Brain Atlas [33] and supplementary materials [34], [35], haplotype, epigenetics and regulation information from UCSC and ENCODE [36], and drug information from Pharmacogenomics Knowledge Base (PharmGKB) [37], Comparative Toxicogenomics Database (CTD) [38] and DrugBank [39], and then parsed the data and rearranged it into well-formatted tables. Transporter substrates were integrated from transporter substrate database (TSdb) [17], whose substrates and drugs of a given transporter were mapped to KEGG LIGAND database. In addition, enriched pathways and diseases information was annotated by KOBAS 2.0 [40].

### Interface Development of Database

**Figure 1:**
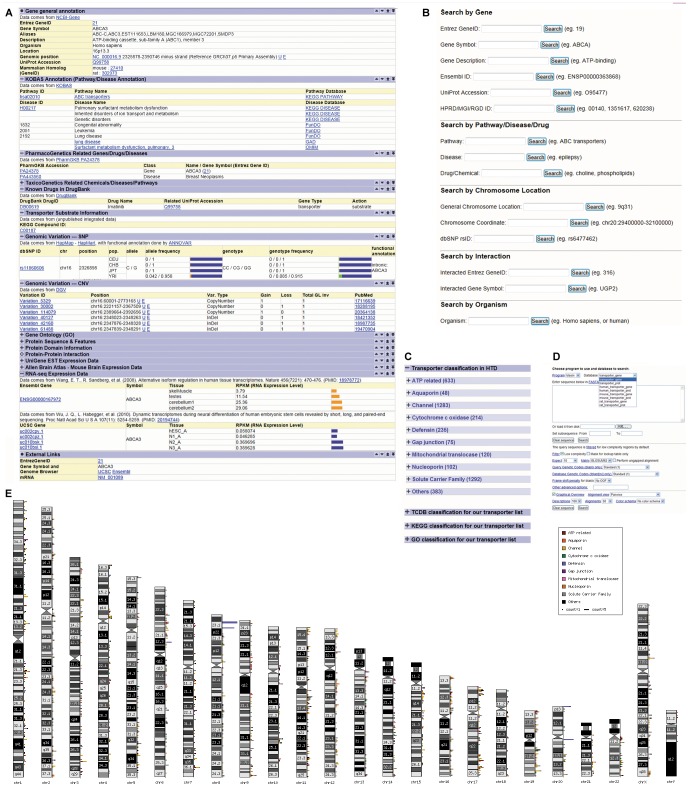
Web interface in HTD. (A) A typical entry for transporter gene (B) Text search; (C) Browsing Human Transporter classification (D) BLAST interface; (E) Chromosome distribution for all the human transporter genes.

The data of HTD is stored in a MySQL relational database [41]–[43]. As shown in Figure S1, to balance the query efficiency and data storage, we integrated multiple dimensional data into some tables, which was easier for update and more flexible to integrate other potential data source. In addition, we mainly used the NCBI Gene ID to crosslink the data tables in our database. The web interface is developed with PHP on Apache server. To achieve a better organization, development and maintenance, we designed the web application into a three-level architecture carefully, including: business layer, which parsed input query and did SQL queries, output function layer, which did sorting and table outputting, and the top web representation layer, which included the frame and styles, and also did some simple redirection. The main interfaces of HTD are user-friendly in browsing the classified transporter genes, querying keywords, and searching sequences for transporter genes (Figure 1).

## Results and Discussion

Combining of automated keywords query, sequence similarity search and manual curation on transporters, we collected 1,555 human non-redundant transporter genes to develop the Human Transporter Database (HTD). To provide a reference, we also collected 1,422 and 1,453 transporter genes for mouse and rat, respectively, in a similar way. A quick exploration showed that 383 (80 protein-coding genes and 303 pseudogenes) human transporter genes (HTGs) were only found in human genome in comparison with transporters from the two rodent genomes, which might be either primate or human specific transporters. To better organize our curated transporter genes, we classified HTGs into ten categories such as “ATP related”, “Channel”, and “Solute Carrier Family” with 98 terms specific for Human Transporters. Based on the statistics on the ten categories (Table 1), 70% of HTGs were from “ATP related”, “Channel”, and “Solute Carrier Family”. We conducted a semi-automatic pipeline to map transporter genes to known transporter classification systems, which mainly included BLAST search and manual checking for TC system, and ID mapping for GO system and KEGG BRITE system. As shown in Figure S2, the number of genes in each TC second-level category was counted after mapping to TC system, and it showed that majority (60%) of HTGs were from 2.A (Porters (uniporters, symporters, antiporters)), 1.A (α-type Channels), and 3.A (P-P-bond-hydrolysis-driven transporters). Indeed, these three TC categories are almost corresponding to ‘Solute Carrier Family’, ‘Channel’ and ‘ATP related’ in our classification system.

**Table 1:** The statistics for transporter genes in ten HTD categories for Human, Mouse and Rat.

Category	Number of transporter genes		
	Human	Mouse	Rat
ATP-binding cassette	222	197	198
Aquaporin	19	13	13
Channel	427	397	442
Cytochrome C oxidase	75	54	63
Defensin	72	102	60
Gap junction	28	23	24
Mitochondrial translocase	39	36	42
Nucleoporin	39	36	27
Solute carrier family	454	404	430
Others+Unclassified	170	160	154
Total	1555	1422	1453

### Information on the Transporter Gene Page

In each gene page, detailed information of general functions, pathways, genetic polymorphisms, phamacogenetics, substrates were listed; and cross-references to their origin databases such as UniProt, PharmGKB, and DrugBank were included (Figure 1A). To better organize information from large scale studies, we collapsed detailed information for protein sequences, features or domain information, protein-protein interactions, and gene expression profiles by default. Clicking on the expanding links ‘+’ of each annotation can bring the graphic views of allele frequency, genotype frequency for SNPs and tissue expression profile of each transporter. Users can expand all the annotation in gene page by clicking “Expand all” button in the top. When exploring each type of annotation, users can click the up/down arrow in the right of the table to reach specific annotation quickly (Figure 1A).

For query speed, the full SNP annotation for a transporter gene was shown in another similar page, which allowed users to filter and only leave exonic or nonsynonymous SNPs and to sort those related SNPs by position, minor allele frequency, difference on population allele frequency, heterozygosity, or functional annotation. The functional annotation for each SNP was mainly based on ANNOVAR [44], including intronic/exonic, synonymous/nonsynonymous, SIFT score [45] and PolyPhen-2 score [46], [47].

### Browsing the Classified Transporter Genes

HTD supports a variety of ways to browse transporter genes, including the hierarchical classification and chromosome distribution. The classification page contains four parts of classification systems, including classification in HTD, TC classification, KEGG BRITE and Gene Ontology, which could be easily chosen by users (Figure 1C). The classification in HTD was mainly based on gene name, domain information and GO annotation, which might correspond to categories in the standard TC system. Each transporter under a specific category is linked to detailed information page of the gene. In addition, the genomic distribution of ten categories of HTGs in our HTD was plotted in 24 chromosomes with different colors (Figure 1E). Users can click on each cytoband in chromosome to access all the transporters in the region.

### Keyword based Search of Transporter Proteins

A quick search box on the top right of each page was useful to search by transporter names or Entrez Gene IDs quickly. Advanced searches were constructed to query HTD by typing their gene name, accession number from NCBI and EBI gene and protein databases and their functional characteristics including chromosome location, interaction partner, biological process, and disease or drug (Figure 1B).

### Sequence based Search of Transporter Proteins

In BLAST page, users can evaluate the transporters with input sequences. The homologs of input sequence are searched among the transporters in HTD using BLAST. The sequence alignment option can be modified with E-value and identity score. This database also provides bulk downloads of all nucleotide and protein sequences in a FASTA format for an advanced local sequence search (Figure 1D).

### Comparison to Other Public Transporter Resources

Our HTD shares 944 transporters with the other four databases, which account for 60.7% in HTD and 80.1% for the total in other four databases. We have included 611 unique membrane HTGs (245 protein-coding genes and 366 pseudogenes) that were not found in previous transporter databases (representing 36.3% of our list). Among the 245 coding genes, there are 89 ion channels (including 9 anoctamins, a group of calcium activated chloride channels), 44 defensins, 20 nuclear pore proteins, 26 cytochrome c oxidases, 11 solute carrier family (SLC) proteins, 9 mitochondrial membrane translocases, 7 ATP-synthases, 5 aquaporins, 2 complement components (C8, and C9), 2 blood group-related proteins, and a scavenger receptor. Thus, our collection includes more ion channels, SLC family proteins and other proteins which are not included in other databases.

As shown in Figure S3, 234 genes from the other four databases were not included in our HTD. In detail, these genes contain 32 fatty acid binding proteins (including 8 lipocalins), 20 BCL family proteins, 17 annexins, 16 genes (including motor proteins) which transport other proteins, 15 GPCR proteins, 14 proteins from membrane-spanning 4-domains subfamily, 13 signal transduction kinases (including MAPK), 13 peroxisomal biogenesis factors, 11 apolipoproteins, 9 signal recognition particles, 5 claudins, 3 lysosomal-associated membrane proteins, 2 cells cytochrome b proteins, 2 odorant binding proteins, and 2 nucleoside kinases. The reasons that we do not include these proteins are as following: 1, not transmembrane transporters, but localizing to cytoplasm or plasma, such as apolipoproteins; 2, some proteins such as motor proteins, which are just associated with cytoplasmic vesicle transporting but not transmembrane transporting; 3, signal transduction proteins such as GPCRs and kinases, which do not participate the transmembrane transporting; 4, other proteins whose substrates locate on or in transmembrane.

To compare with TCDB, we downloaded all the human transporters from TCDB (http://www.tcdb.org/hgnc_explore.php) and did one by one gene symbol comparison. We found additional transporters that are not in TCDB, e.g. AQP3 and AQP7. If we include human pseudogene, there are 952 HTD unique entries. If we exclude pseudogene, there are still 579 HTD unique genes not including in TCDB. The complete mapping information between our HTD and TCDB can be found in our web site (http://htd.cbi.pku.edu.cn/download/htd2tcdb.xls). In addition, we also built the phylogenetic trees for all the categories based on our HTD classification system. All the multiple alignment results can be found in our updated web site (http://htd.cbi.pku.edu.cn/download/multiple_alignment_and_ML_tree.zip) that will help users to gain more insight for the evolutionary aspect of each transporter categories. Evolutionarily, HTD is complementary to TCDB.

### Statistical Analyses on Expression, Variation, Function, Disease Profiles

Based on our collected heterogeneous data, we conducted systems biology data integration which might remove bias resulting from any single technology platform and provide additional insight into the genetic etiology not observed by any individual study [48], [49]. The expression level changes of transporters could cause wide effects on compound and drug metabolism. In helping users to gain an overview for the gene expression pattern of a given transporter, we integrated publicly available gene expression profiling data of the transporters. Overall, the expression data integration was mainly based on ID mapping. The EST expression levels in different tissues were integrated from NCBI UniGene, which could be directly linked to NCBI Entrez Gene ID. Mouse brain region expression profiles were from Allen Brain Atlas [33], which were mapped to human Gene ID based on homology information from NCBI HomoloGene. The RNA-seq expression data was extracted from supplementary materials [34], [35], which were followed by ID mapping from Ensembl Gene ID or UCSC Gene ID to Entrez Gene ID. Transporters with drug targets were reported more commonly to express in many tissues such as intestine, liver, kidney, and brain for drug absorption and excretion. Based on the brain gene expression data from Allen Brain Atlas, we compared the expression levels of transporters with non-transporter genes. We applied Fisher’s exact test on a 2-by-2 contingency table counting the gene number of transporter or non-transporter genes with low (< = 5 in 100 scale) or high (>5 in 100 scale) expression level. In almost all brain regions, the proportion of mouse transporter genes with low expression level is significantly smaller than non-transporter genes. This indicated that transporter genes overall express higher than other genes in brain regions (Figure S4). Additionally, based on RNA-seq data for human tissues, we observed similar expression pattern in various brain regions when comparing to other tissues or cell lines (Figure S5) [34].

**Figure 2:**
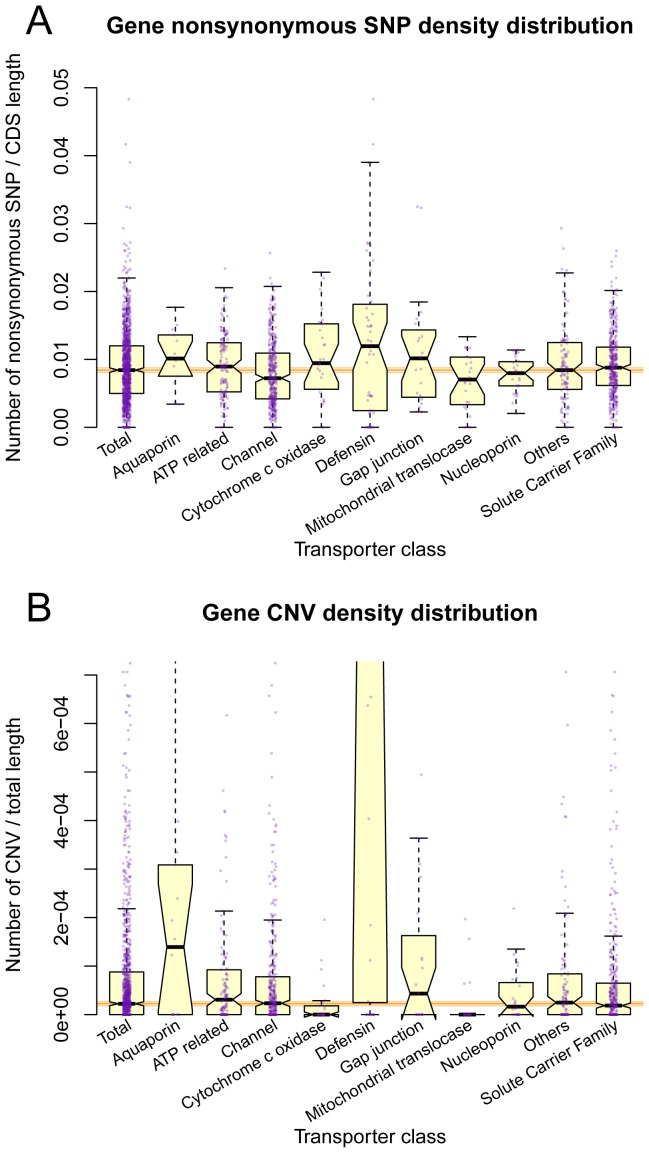
Distribution of nonsynonyous SNP and CNV density on ten categories of transporter genes in HTD. The x-axis shows the ten transporter categories, and y-axis shows the corresponding value: (A) the density of nonsynonymous SNPs, normalized by dividing CDS length, (B) the density of CNVs, normalized by dividing gene total length. Both subfigures are notched boxplot along with scattered real sample points in purple. The thick band inside the box is the median, and the bottom and top of the box are the first quantile (Q1) and the third quantile (Q3). The ends of the whiskers represents data within 1.5 *IQR ( = Q3–Q1) from the lower quantile (Q1) or the upper quantile (Q3). The notch is always symmetric around the median, with deviation from median by 1.58 *IQR/sqrt(n), where n is the sample size. The notch approximately shows the confidence interval of median, so that if the notches of two boxes do not overlap, their medians are usually significantly different. Three horizontal orange lines behind the boxes show the median and notch range of the “Total” box.

The genetic polymorphisms in transporters often have direct or adverse effects on the pharmacokinetics, drug-drug interactions, and personalized drug treatments [50]. The integration of genetics, disease, and drug information related to transporters provides an overview for the therapeutic safety and efficacy of drugs in various diseases. Based on population SNP information from dbSNP and HapMap, 1,279 genes (82.3%) from 1,555 human transporters overlapped 1,201,561 SNPs, in which 35,358 SNPs are exonic and 19,183 are nonsynonymous. When focusing on nonsynonymous SNPs, the HTGs from “Cytochrome c oxidase”, “Defensin”, and “Mitochondrial translocase” contained significantly less nonsynonymous SNPs in comparison with other transporter genes (Figure S6A). To control the potential influence of CDS length, which was shown different between categories (Figure S6B), we calculated the SNP density by dividing gene CDS length. After normalization, the average nonsynonymous SNP density for “Defensin” was marginally significantly higher than others (Wilcoxon rank sum test, *p*-value = 0.078), and “Channel” has lower SNP density (*p*-value = 2.5e-5) (Figure 2A). Copy-number variations (CNVs) refer a structure variation resulting gain or loss of copies of one or more sections of chromosome. Based on the integrated CNV data from DGV database, 855 genes (55.0%) from 1,555 human transporters were overlapped with known CNV regions. With the same analysis approach, after controlling gene total length (Figure S6C, D), CNV density was found significantly higher in “Defensin” (*p*-value = 7.0e-12), and lower in “Cytochrome c oxidase” (*p*-value = 3.1e-03) and “Mitochondrial translocase” (*p*-value = 1.5e-03) (Figure 2B). These results might suggest that “Defensin” genes were subjected to weaker negative selection than other transporter genes.

Further functional enrichment analyses showed that 1,555 HTGs were enriched in various cellular processes. Some of the highlights include oxidative phosphorylation, cardiac muscle contraction, Parkinson’s disease, vibrio cholerae infection, mineral absorption, collecting duct acid secretion, synaptic vesicle cycle, ABC transporter, Alzheimer’s disease, and bile secretion (all FDR corrected *p*-value <1e-16). Furthermore, we found that HTGs were mostly enriched in neural disease, drug abuse, and other metabolic disorders such as epilepsy, sudden infant death syndrome, long QT syndrome, and congenital disorders of ion transport and metabolism (Table 2). With manually integrated information according to OMIM, GAD, and MeSH, 215 HTGs were related to 21 diseases categories. There were 101 HTGs that are related to “nervous system diseases”, 79 HTGs related to “congenital, hereditary, and neonatal diseases and abnormalities”, 58 HTGs related to “nutritional and metabolic diseases”, and 43 HTGs related to “cardiovascular diseases” (Figure S7A). To get an overview of natural polymorphisms on disease associated transporters we counted the number of non-synonymous mutations and CNVs on the HTGs and normalized by length. When comparing to all HTGs, most disease-related HTGs tended to have longer CDS length, among which HTGs related to “congenital, hereditary, and neonatal diseases and abnormalities” and “nutritional and metabolic diseases” were found to have significantly longer CDS (Wilcoxon rank sum test, *p*-value = 8.5e-9 and 2.6e-03) (Figure S7B). HTGs related to “bacterial infections and mycoses” and “respiratory tract diseases” were found to have significantly higher nonsynonymous SNP density (*p*-value = 7.3e-03 and 8.6e-03), while HTGs related to “mental disorders” tended to have lower nonsynonymous SNP density (*p*-value = 0.086) (Figure S7C). Most of 21 disease categories showed similar distribution on the density of CNVs involved with HTGs (Figure S7D).

**Table 2:** Top ten enriched diseases of human transporter genes.

Disease	Database	P Value	Q Value*
Epilepsy	GAD	1.68E-14	7.06E-13
Sudden infant death syndrome	FunDO	5.26E-10	1.41E-08
Other nervous and sensory system diseases	KEGG DISEASE	2.53E-08	5.20E-07
Long QT syndrome	FunDO	8.40E-08	1.61E-06
Congenital disorders of ion transport and metabolism	KEGG DISEASE	3.09E-07	5.57E-06
Drug abuse	FunDO	3.52E-07	6.11E-06
Atrial fibrillation	KEGG DISEASE	4.23E-07	6.92E-06
Brugada syndrome (BRS)	KEGG DISEASE	4.23E-07	6.92E-06
Serum uric acid	GAD	8.96E-07	1.39E-05
Nervous system diseases	KEGG DISEASE	2.05E-06	2.88E-05

*Benjamini-Hochberg Corrected P Value.

### Integrated Analyses on Variations and Drugs of Human Transporters

As HTGs may play the important roles in drug metabolism, we integrated pharmacogenetics and drug information from PharmGKB, CTD, and DrugBank, which was also mainly based on NCBI Gene ID mapping. These databases told about the relationship between a drug or chemical and a gene. Due to the statistical power on the number of related genes for a chemical, here we only showed the analysis results based on CTD annotation data. In Figure S8A, the numbers of related HTGs on top 30 types of chemicals with at least 60 related HTGs were plotted. The result indicated that HTGs are highly related to the drugs such as Acetaminophen (pain reliever), Phenobarbital (anticonvulsant), and Valproic Acid (anticonvulsant). We further calculated the nonsynonymous SNP density and CNV density for these groups of HTGs, and found that “Ozone”-related transporter genes had significantly longer CDS than other transporter genes (Wilcoxon rank sum test, *p*-value = 1.1e-03) (Figure S8B), and “Aflatoxin B1”-related transporter genes had significantly higher nonsynonymous SNP density than others (*p*-value = 7.8e-03) (Figure S8C). When checking about CNV, “Ozone”-related and “bisphenol A”-related HTGs tended to have higher CNV density, but not significantly (*p*-value = 0.124 and 0.314), while “Sodium Selenite”-related HTGs had lower CNV density, which was marginally significant (*p*-value = 0.057) (Figure S8D).

## Conclusion and Future Direction

HTD is a comprehensive knowledge-base of Human Transporter resource with extensive pharmacogenetic and genomic annotations. HTD will aid personalized drug development in keeping pace with high-throughput NGS data related to transporters and be updated periodically. Additionally to those integration issues, new tools like literature mining on transporter substrate relationship will be developed to enhance specificity in Human Transporter annotations, and more convenient online analytic tools will be developed to assist online data visualization.

## Supporting Information

Figure S1 **Diagram of Internal Table Structure of HTD.** The table names are shown in the cells with blue background. Those cells filled in red are the major cross-link fields between tables; the two filled in yellow (HomoloGene table) are cross-links for multiple species.
(PDF)

Figure S2 **Data statistics based on TC system.** The transporter genes were classified into TC system by BLAST and manually checked for three species. The amounts in TC system level 2 categories are shown in the graph (The level 2 categories are: 1.A: α-Type Channels; 1.B: β-Barrel Porins; 1.C: Pore-Forming Toxins (Proteins and Peptides); 1.F: Vesicle Fusion Pores; 1.H: Paracellular Channels; 2.A: Porters (uniporters, symporters, antiporters); 3.A: P-P-bond-hydrolysis-driven transporters; 3.D: Oxidoreduction-driven transporters; 4.C: Acyl CoA ligase-coupled transporters; 5.B: Transmembrane 1-electron transfer carriers; 8.A: Auxiliary transport proteins; 9.A: Recognized transporters of unknown biochemical mechanism; 9.B: Putative transport proteins).
(PDF)

Figure S3 **A venn diagram comparison of human transporter genes in HTD with other four popular transporter databases.**
(PDF)

Figure S4 **Gene expression patterns in different brain regions.** The expression patterns of human transporter genes (in red) were shown in different mouse brain areas along with all genes (in black) as background based on mouse brain region expression profiles described in Allen Brain Atlas data. The *p*-values from Fisher’s exact tests demonstrate the decreased proportion of low expression level (scaled expression level 0∼5) of transporter genes compared with all background genes.
(PDF)

Figure S5 **The Gene expression patterns in different tissues.** The expression patterns of human transporter genes (in red) were shown in different tissues along with all genes (in black) as background based on the data from one RNA-seq paper [34]. The *p*-values from Fisher’s exact tests demonstrate the decreased proportion of low expression level (RPKM 0∼5) of transporter genes compared with all background genes.
(PDF)

Figure S6 **Distribution of SNP, CNV count and gene length on ten categories of transporter genes in HTD.** The x-axis shows the ten transporter categories, and y-axis shows the corresponding value: (A) the number of nonsynonymous SNPs on gene CDS region, (B) gene CDS length, (C) the number of CNVs overlapping the total-length gene, (D) gene total length. All four subfigures are standard notched boxplot with scattered real sample points in purple. The thick band inside the box is the median, and the bottom and top of the box are the first quantile (Q1) and the third quantile (Q3). The ends of the whiskers represents data within 1.5 *IQR ( = Q3–Q1) from the lower quantile (Q1) or the upper quantile (Q3). The notch is always symmetric around the median, with deviation from median by 1.58 *IQR/sqrt(n), where n is the sample size. The notch approximately shows the confidence interval of median, so that if the notches of two boxes do not overlap, their medians are usually significantly different. Three horizontal orange lines show the median and notch range of the “Total” box.
(PDF)

Figure S7 **Distribution of gene count, gene length, SNP and CNV density on transporters related to different disease categories.** The x-axis shows 21 disease categories, and y-axis shows the corresponding value: (A) the number of related genes for each disease category, (B) gene CDS length, (C) the density of nonsynonymous SNPs on gene CDS length, (D) the density of CNVs on gene total length. Except the first barplot shows the number of genes related to each disease category, the other three subfigures are standard notched boxplot with scattered real sample points in purple. Three horizontal orange lines show the median and notch range of the “Total” box. The meaning of notched boxplot representation is described in figure legends for Figure S6.
(PDF)

Figure S8 **Distribution of gene count, gene length, SNP and CNV density on transporters related to some chemicals.** The x-axis shows top 30 chemicals related with most transporter genes, and y-axis shows the corresponding value: (A) the number of related genes for each disease category, (B) gene CDS length, (C) the density of nonsynonymous SNPs on gene CDS length, (D) the density of CNVs on gene total length. Except the first barplot shows the number of genes related to a chemical, the other three subfigures are standard notched boxplot with scattered real sample points in purple. Three horizontal orange lines show the median and notch range of the “Total” box. The meaning of notched boxplot representation is described in figure legends for Figure S6.
(PDF)

Table S1 **Curated keywords for literature searching and corresponding category in HTD.**
(DOCX)

Table S2 **Annotation entry statistics for 1555 human transporter genes.**
(DOC)
