# Correction: Human Transporter Database: Comprehensive Knowledge and Discovery Tools in the Human Transporter Genes

**DOI:** 10.1371/journal.pone.0098396

**Published:** 2014-05-16

**Authors:** The PLOS ONE Staff

Table S2 contains track changes in both the XML and PDF versions of the article. Please see the corrected Table below.

## Supporting Information

Table S2 **Annotation entry statistics for 1555 human transporter genes.**
(DOC)
