# Supplementary material for: Human Transporter Database: Comprehensive Knowledge and Discovery Tools in the Human Transporter Genes
Source: PLoS One. 2014 Feb 18;9(2):e88883. doi: 10.1371/journal.pone.0088883 (PMC3928311; doi:10.1371/journal.pone.0088883)
Supplement: Figure S1 — Diagram of Internal Table Structure of HTD. The table names are shown in the cells with blue background. Those cells filled in red are the major cross-link fields between tables; the two filled in yellow (HomoloGene table) are cross-links for multiple species. (PDF) [file pone.0088883.s001.pdf]

GeneID_list
GeneID

NCBI_Gene
GeneID
GeneSymbol
Aliases
Description
Organism
Location
Ensembl
HPRD
OMIM
GenomeHeading
GenomeAc
GenomeVer
Genome_gi
GenomeFrom
GenomeTo
GenomeOrient
mRNA_Ac
protein_Ac
UniProt_SwissProt
UniGene
MGI
RGD
Primary_UniProtAC

HPRD_ID_mapping
hprd_id
geneSymbol
nucleotide_accession
protein_accession
entrezgene_id
omim_id
swissprot_id
main_name

HPRD_PPI
interactor_1_geneSymbol
interactor_1_hprd_id
interactor_1_refseq_id
interactor_2_geneSymbol
interactor_2_hprd_id
interactor_2_refseq_id
experiment_type
reference_id

UniProt
UniProtID
UniProtAc
Description
GeneName
Organism
EMBL
IPI
PIR
RefSeq
UniGene
PDB
STRING
TCDB
PRIDE
Ensembl
GeneID
GeneCards
MIM
PharmGKB
BioCyc
Reactome
GO
Feature
Sequence

InterPro_domain
GeneID
UniProtAC
InterProAC
InterProName
Position
otherID
otherName

HomoloGene
HomoloGene_GroupID
TaxonomyID
GeneID
GeneSymbol
ProteinGI
ProteinAccession

Allen_Brain_Atlas
EntrezGeneID
GeneID
GeneName
GeneSymbol
Organism
avgDensity
avgLevel
structureLabel
structureName

UniGene_EST
UniGeneID
pool_class
pool_name
gene_EST
total_EST

RNAseq_1_ID_mapping
entrez
ensembl

RNAseq_1_RPKM
Gene
UHRLowcov
brainLowcov
adipose
brain
breast
colon
heart
liver
lymphNode
skelMuscle
testes
cerebellum1
cerebellum2
cerebellum3
cerebellum4
cerebellum5
cerebellum6
MCF7
BT474
HME
MB435
T47D
Symbol
Description

RNAseq_2_ID_mapping
Entrez
UCSC

RNAseq_2_expression
UCSC_Known_Gene
Gene_Symbol
hESC_A
N1_A
N2_A
N3_A
Pattern

ENCODE_TFBS_info
chr
chr_start
chr_end
TF_name
score
cell_types
TfbsID

ENCODE_gene2TFBS
transporter_GeneID
TfbsID

ENCODE_TFBS2SNP
TfbsID
rsID

dbSNP_HapMap_add_ANNOVAR
rsId
alleles
heterozygosity, S.E.
MAF, allele count
chr, pos, orient
HapMap ref/other allele/genotype
ASW allele/genotype count
CEU allele/genotype count
CHB allele/genotype count
CHD allele/genotype count
GIH allele/genotype count
JPT allele/genotype count
LWK allele/genotype count
MEK allele/genotype count
MKK allele/genotype count
TSI allele/genotype count
YRI allele/genotype count
transporter_GeneID
allele_pop_diff_log10_pvalue
diff_ref_proportion
variation_function
exonic_variation_function
sift_score
pp2_score

dbSNP_HapMap_gene_snp_map
transporter_GeneID
chr
rsId

DGV_mapGeneID2variation
DGV_mapGeneID2indel
EntrezGeneID
VariationID

DGV_variation
DGV_indel
VariationID
Landmark
Chr
Start
End
VariationType
LocusChr
LocusStart
LocusEnd
Reference
PubMedID
Method_platform
Gain
Loss
TotalGainLossInv
Frequency
SampleSize
Genes

PharmGKB_gene_genes
entrezGeneID
pharmgkbAccession
geneSymbol
pharmgkbAccession2
geneSymbol2
entrezGeneID2

PharmGKB_gene_drugs
PharmGKB_gene_diseases
entrezGeneID
pharmgkbAccession
geneSymbol
pharmgkbAccession2
name2

DrugBank
DrugBank_DrugID
DrugName
Type
DrugBank_GeneID
Action
GeneSymbol
UniProtAC
UniProtID
NCBI_GeneID

TSdb_substrate
UniProt_AC
CoupoundID_KEGG

CTD_chem_gene
ChemicalName
ChemicalID
CasRN
GeneSymbol
GeneID
Organism
OrganismID
Interaction
InteractionActions
PubMedIDs

CTD_gene_disease
GeneSymbol
GeneID
DiseaseName
DiseaseID
DirectEvidence
InferenceChemicalName
InferenceScore
OmimIDs
PubMedIDs

CTD_gene_pathways
GeneSymbol
GeneID
PathwayName
PathwayID

KOBAS_genes_pathways
gene_id
puid

KOBAS_pathways
puid
pathway_id
pathway_db
species_abbr
pathway_class
pathway_name

KOBAS_genes_disease
gene_id
duid

KOBAS_diseases
duid
disease_id
disease_db
species_abbr
disease_class
disease_name
