# Supplementary figures and images for: Human Transporter Database: Comprehensive Knowledge and Discovery Tools in the Human Transporter Genes
Source: PLoS One. 2014 Feb 18;9(2):e88883. doi: 10.1371/journal.pone.0088883 (PMC3928311; doi:10.1371/journal.pone.0088883)

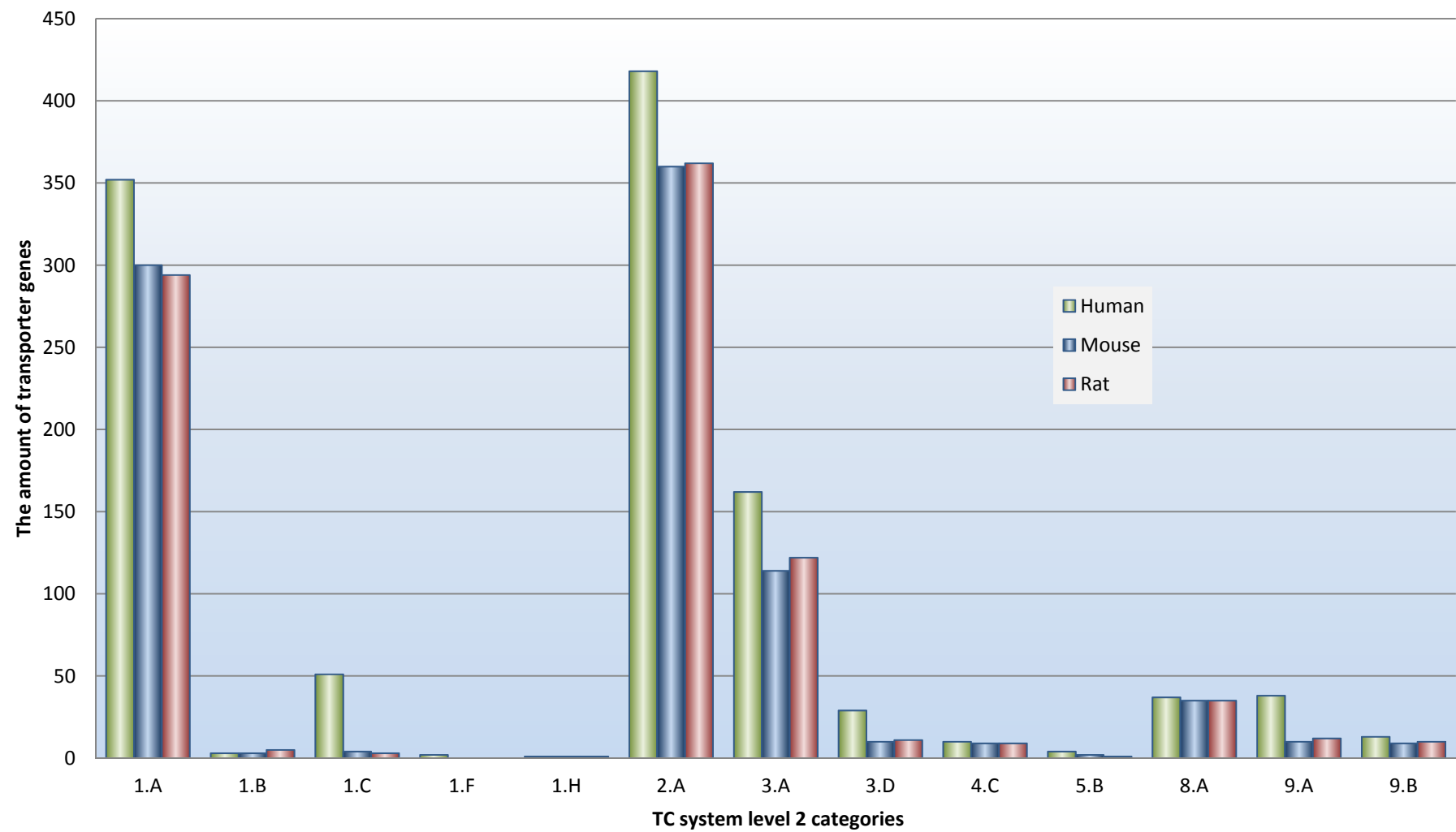

Supplement: Figure S2 — Data statistics based on TC system. The transporter genes were classified into TC system by BLAST and manually checked for three species. The amounts in TC system level 2 categories are shown in the graph (The level 2 categories are: 1.A: α-Type Channels; 1.B: β-Barrel Porins; 1.C: Pore-Forming Toxins (Proteins and Peptides); 1.F: Vesicle Fusion Pores; 1.H: Paracellular Channels; 2.A: Porters (uniporters, symporters, antiporters); 3.A: P-P-bond-hydrolysis-driven transporters; 3.D: Oxidoreduction-driven transporters; 4.C: Acyl CoA ligase-coupled transporters; 5.B: Transmembrane 1-electron transfer carriers; 8.A: Auxiliary transport proteins; 9.A: Recognized transporters of unknown biochemical mechanism; 9.B: Putative transport proteins). (PDF) [file pone.0088883.s002.pdf]

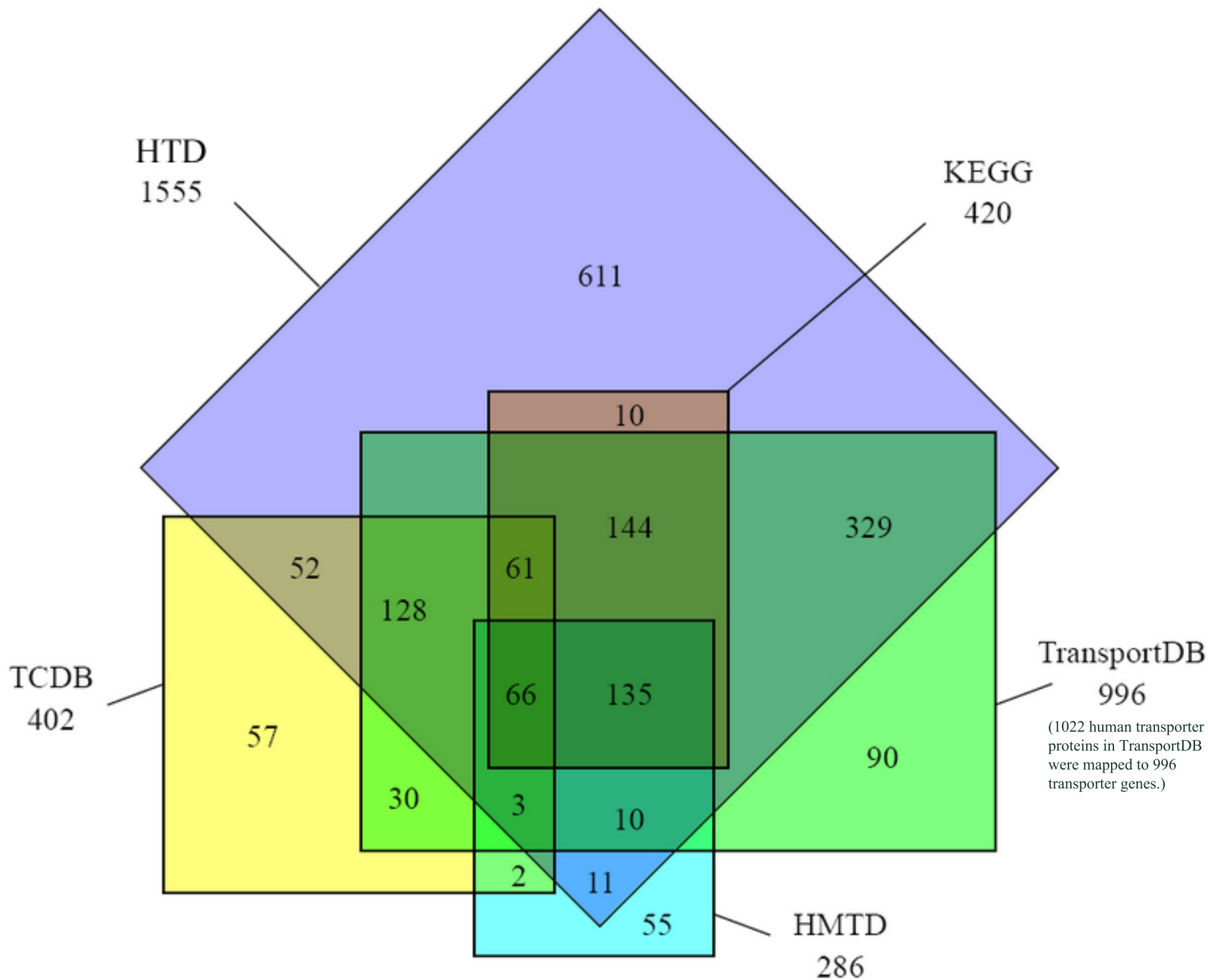

Supplement: Figure S3 — A venn diagram comparison of human transporter genes in HTD with other four popular transporter databases. (PDF) [file pone.0088883.s003.pdf]

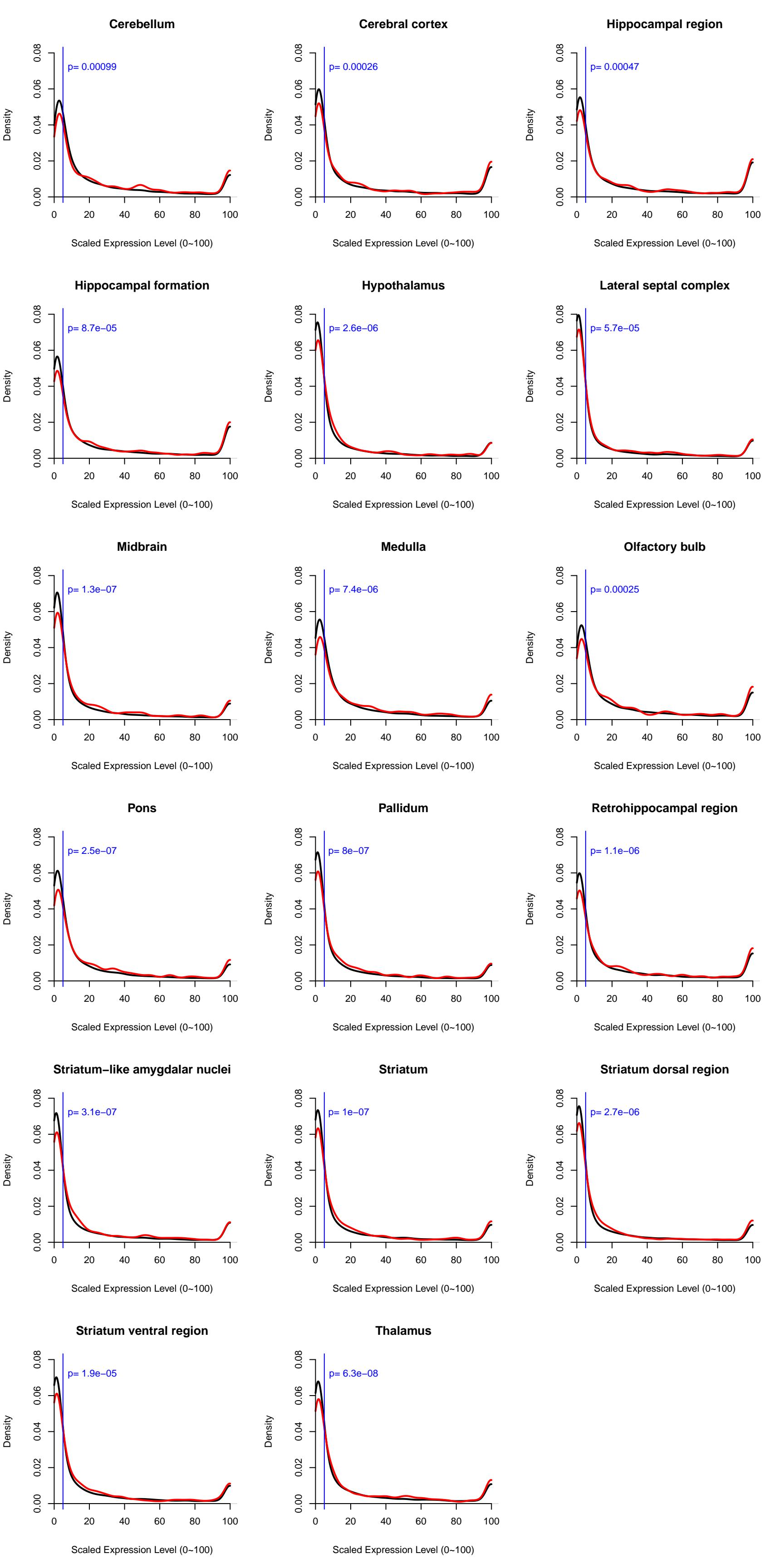

Supplement: Figure S4 — Gene expression patterns in different brain regions. The expression patterns of human transporter genes (in red) were shown in different mouse brain areas along with all genes (in black) as background based on mouse brain region expression profiles described in Allen Brain Atlas data. The p-values from Fisher’s exact tests demonstrate the decreased proportion of low expression level (scaled expression level 0∼5) of transporter genes compared with all background genes. (PDF) [file pone.0088883.s004.pdf]

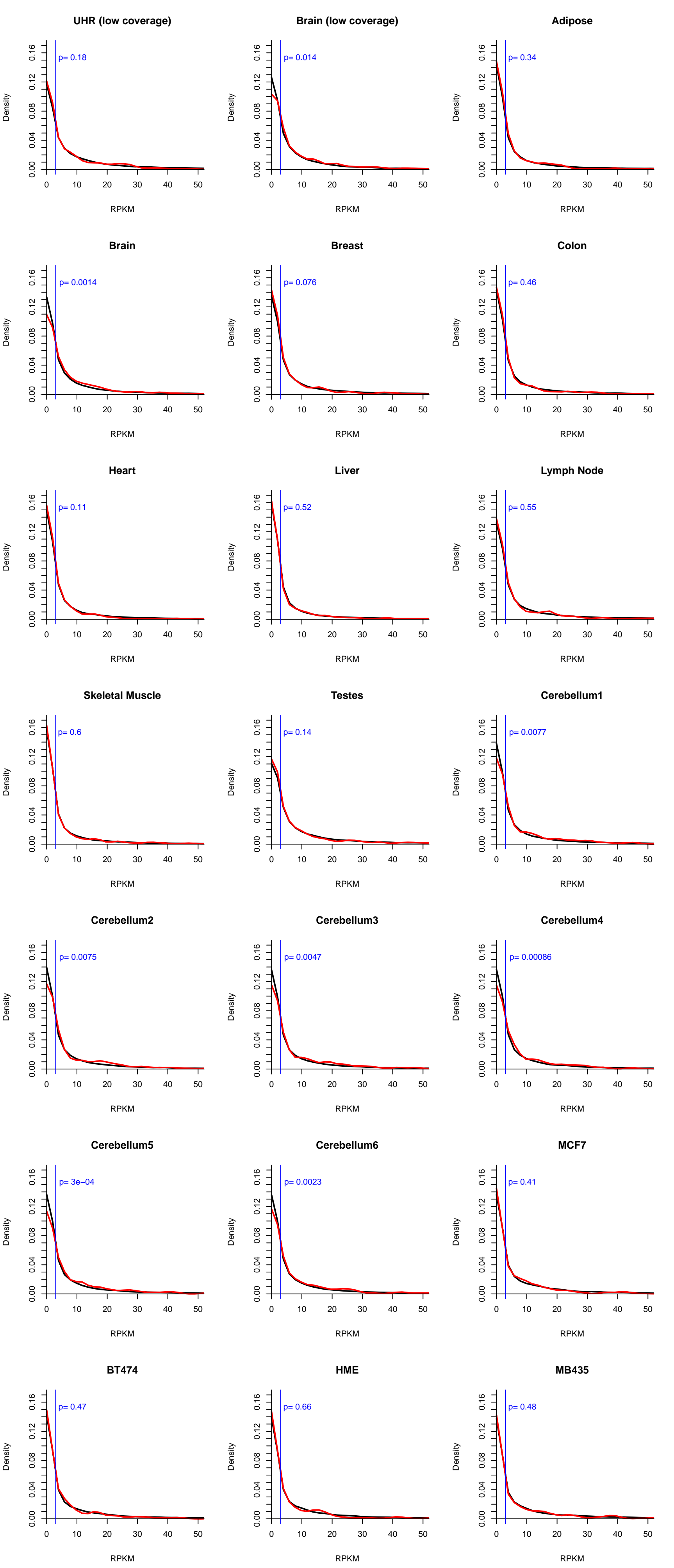

Supplement: Figure S5 — The Gene expression patterns in different tissues. The expression patterns of human transporter genes (in red) were shown in different tissues along with all genes (in black) as background based on the data from one RNA-seq paper [34]. The p-values from Fisher’s exact tests demonstrate the decreased proportion of low expression level (RPKM 0∼5) of transporter genes compared with all background genes. (PDF) [file pone.0088883.s005.pdf]

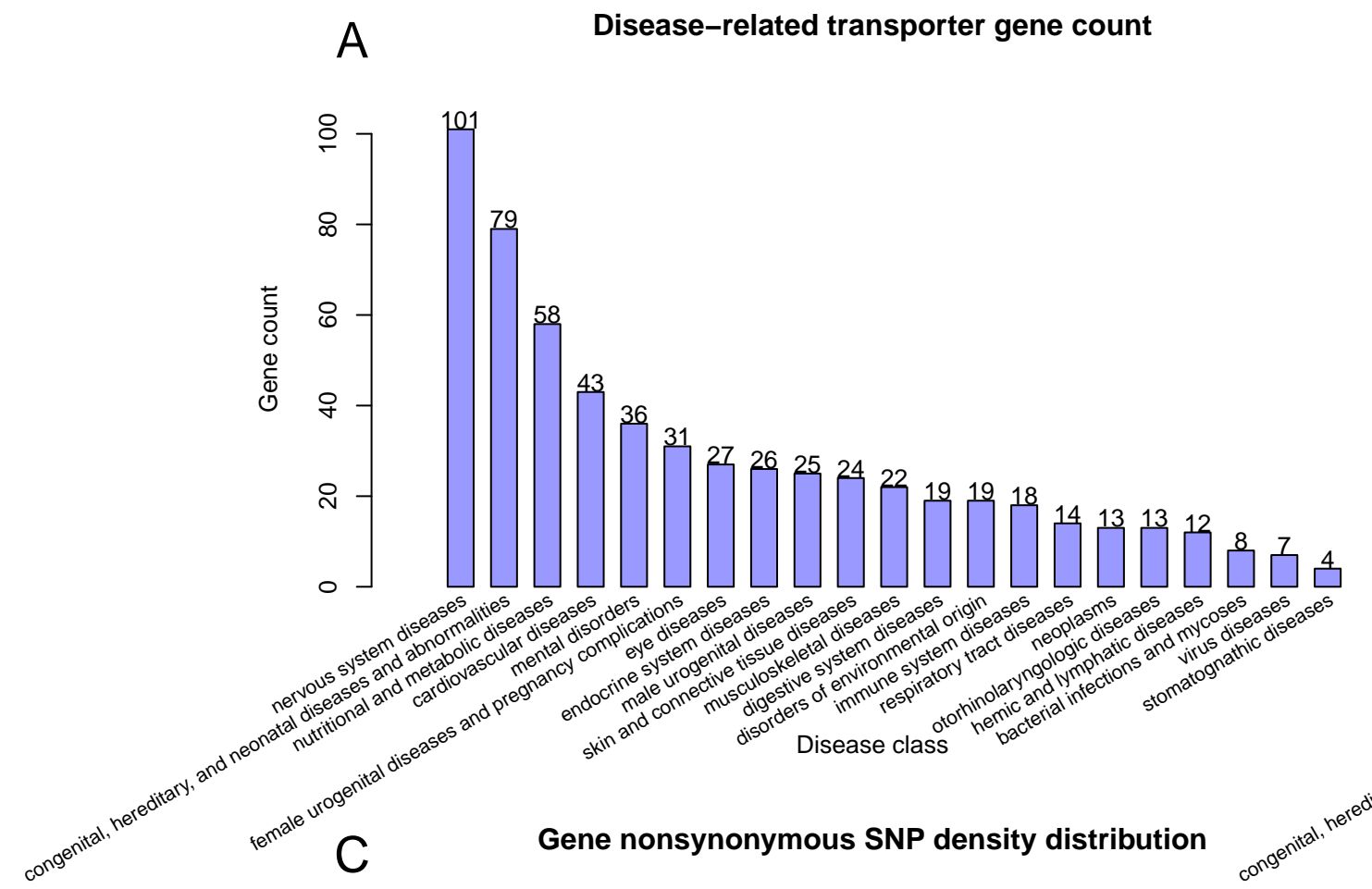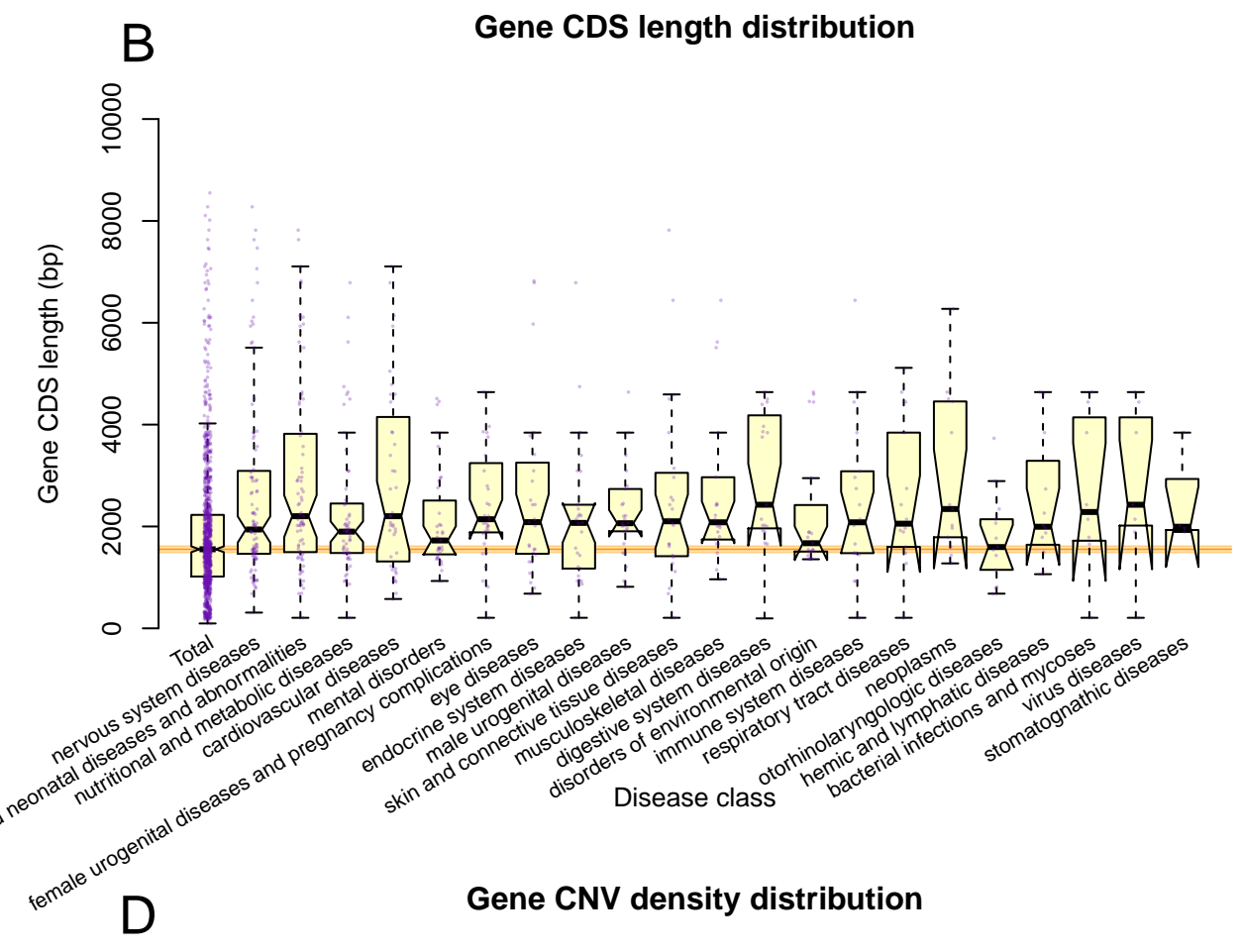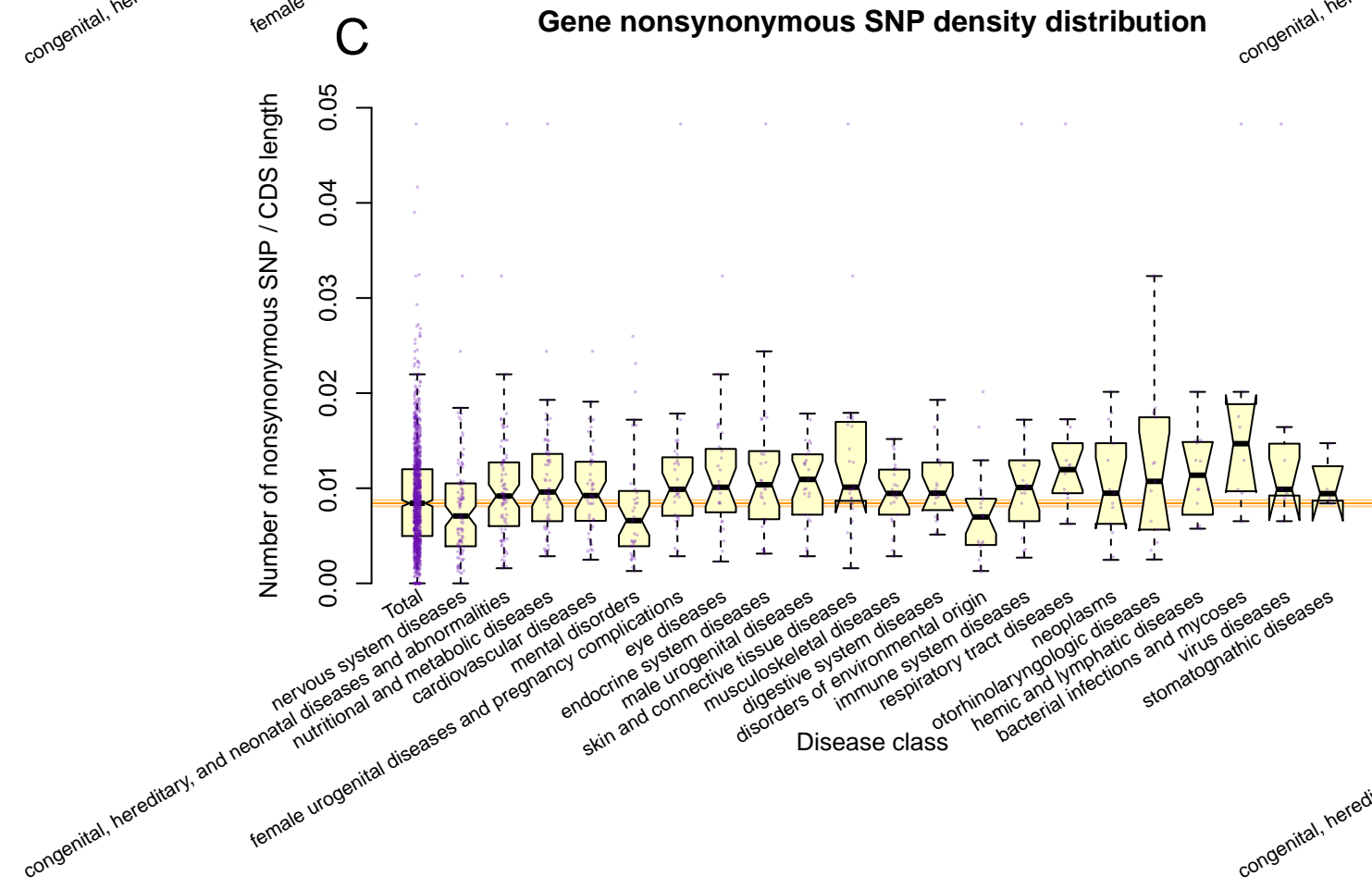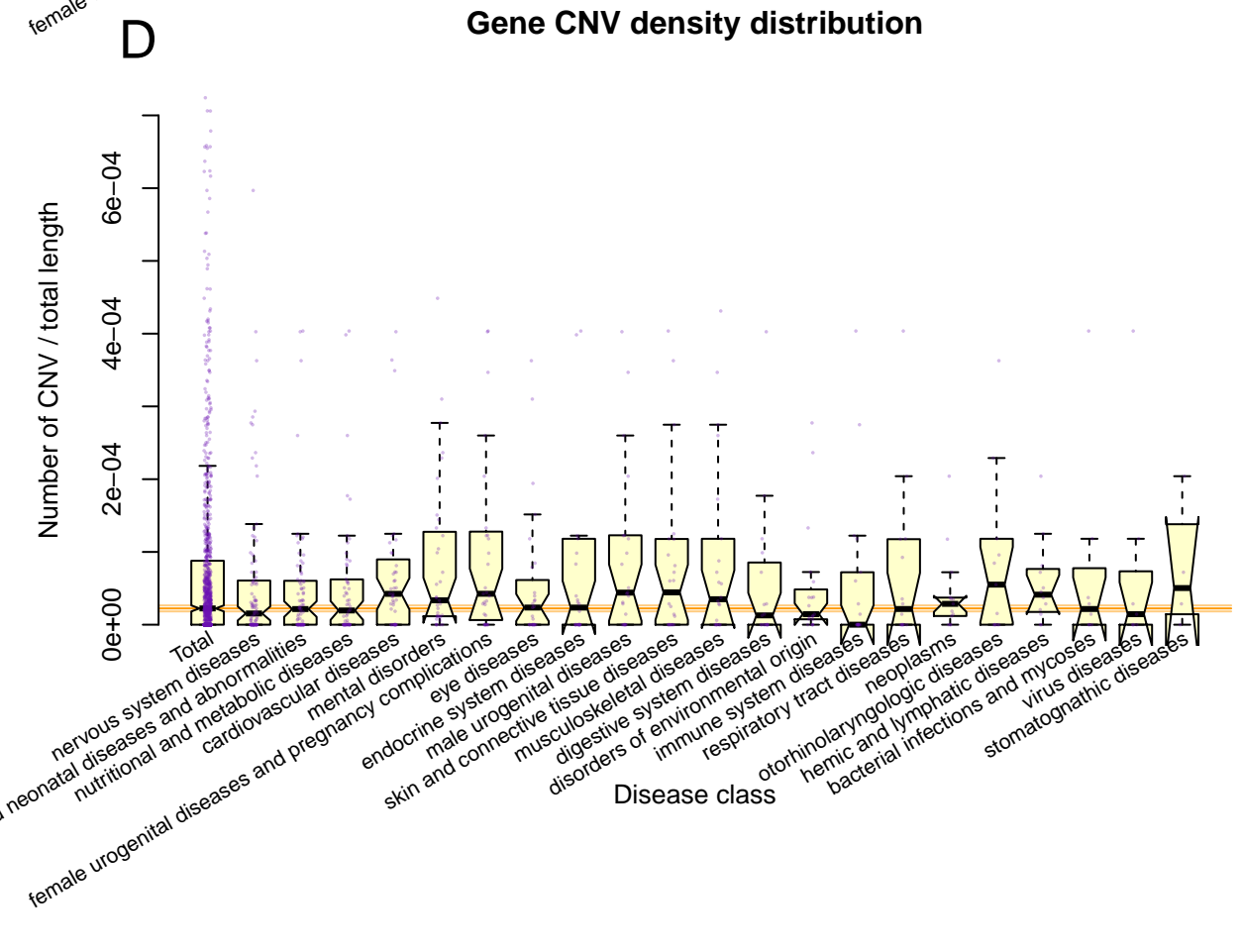

Supplement: Figure S7 — Distribution of gene count, gene length, SNP and CNV density on transporters related to different disease categories. The x-axis shows 21 disease categories, and y-axis shows the corresponding value: (A) the number of related genes for each disease category, (B) gene CDS length, (C) the density of nonsynonymous SNPs on gene CDS length, (D) the density of CNVs on gene total length. Except the first barplot shows the number of genes related to each disease category, the other three subfigures are standard notched boxplot with scattered real sample points in purple. Three horizontal orange lines show the median and notch range of the “Total” box. The meaning of notched boxplot representation is described in figure legends for Figure S6. (PDF) [file pone.0088883.s007.pdf]
