# Supplementary material for: Human Transporter Database: Comprehensive Knowledge and Discovery Tools in the Human Transporter Genes
Source: PLoS One. 2014 Feb 18;9(2):e88883. doi: 10.1371/journal.pone.0088883 (PMC3928311; doi:10.1371/journal.pone.0088883)
Supplement: Figure S6 — Distribution of SNP, CNV count and gene length on ten categories of transporter genes in HTD. The x-axis shows the ten transporter categories, and y-axis shows the corresponding value: (A) the number of nonsynonymous SNPs on gene CDS region, (B) gene CDS length, (C) the number of CNVs overlapping the total-length gene, (D) gene total length. All four subfigures are standard notched boxplot with scattered real sample points in purple. The thick band inside the box is the median, and the bottom and top of the box are the first quantile (Q1) and the third quantile (Q3). The ends of the whiskers represents data within 1.5 *IQR ( = Q3–Q1) from the lower quantile (Q1) or the upper quantile (Q3). The notch is always symmetric around the median, with deviation from median by 1.58 *IQR/sqrt(n), where n is the sample size. The notch approximately shows the confidence interval of median, so that if the notches of two boxes do not overlap, their medians are usually significantly different. Three horizontal orange lines show the median and notch range of the “Total” box. (PDF) [file pone.0088883.s006.pdf]

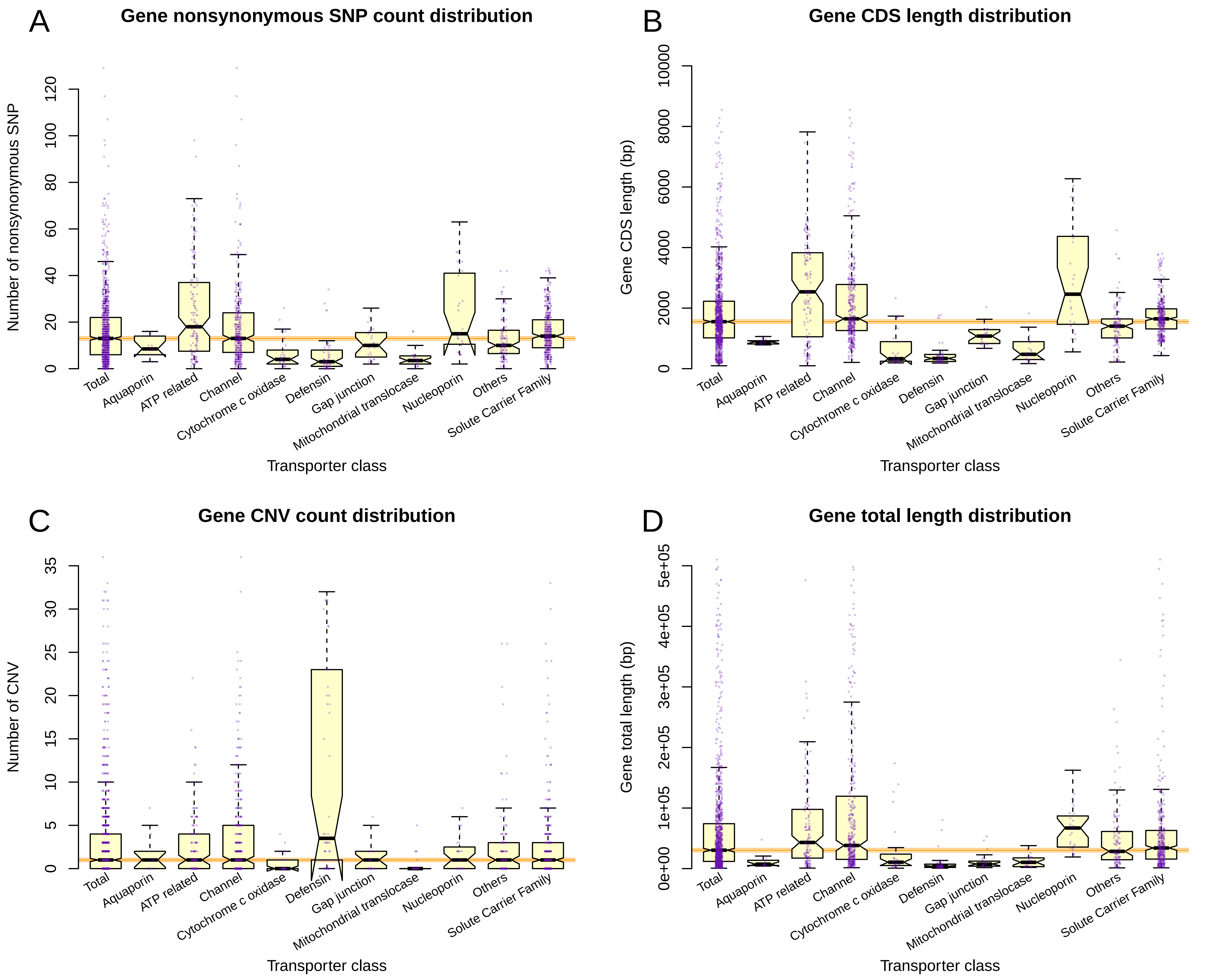
A
Gene nonsynonymous SNP count distribution
Number of nonsynonymous SNP
0
20
40
60
80
100
120
Total
Aquaporin
ATP related
Channel
Cytochrome c oxidase
Defensin
Gap junction
Mitochondrial translocase
Nucleoporin
Others
Solute Carrier Family
Transporter class
B
Gene CDS length distribution
Gene CDS length (bp)
0
2000
4000
6000
8000
10000
Transporter class
C
Gene CNV count distribution
Number of CNV
0
5
10
15
20
25
30
35
Transporter class
D
Gene total length distribution
Gene total length (bp)
0e+00
1e+05
2e+05
3e+05
4e+05
5e+05
Transporter class
