# Supplementary material for: Human Transporter Database: Comprehensive Knowledge and Discovery Tools in the Human Transporter Genes
Source: PLoS One. 2014 Feb 18;9(2):e88883. doi: 10.1371/journal.pone.0088883 (PMC3928311; doi:10.1371/journal.pone.0088883)
Supplement: Figure S8 — Distribution of gene count, gene length, SNP and CNV density on transporters related to some chemicals. The x-axis shows top 30 chemicals related with most transporter genes, and y-axis shows the corresponding value: (A) the number of related genes for each disease category, (B) gene CDS length, (C) the density of nonsynonymous SNPs on gene CDS length, (D) the density of CNVs on gene total length. Except the first barplot shows the number of genes related to a chemical, the other three subfigures are standard notched boxplot with scattered real sample points in purple. Three horizontal orange lines show the median and notch range of the “Total” box. The meaning of notched boxplot representation is described in figure legends for Figure S6. (PDF) [file pone.0088883.s008.pdf]

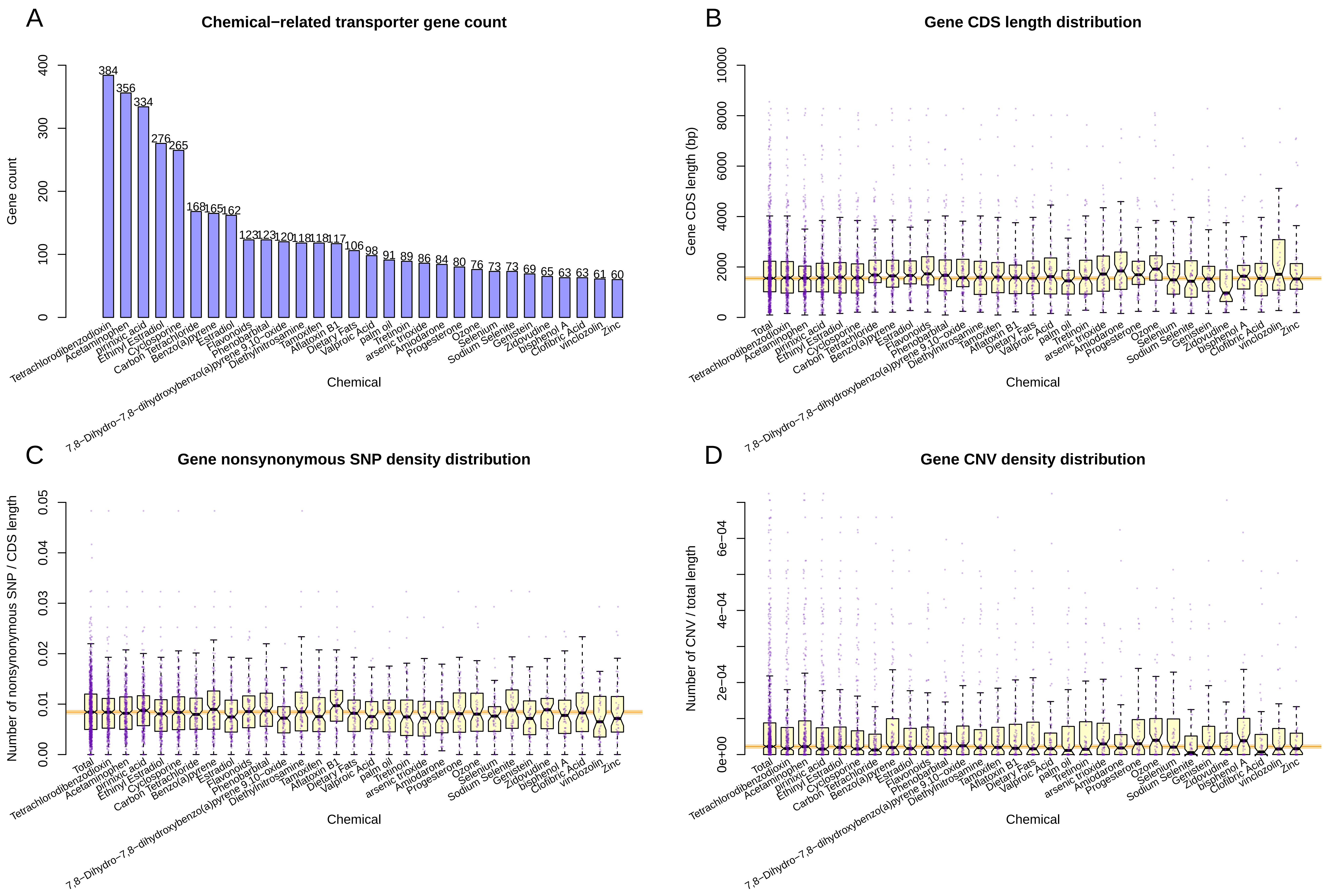
A
Chemical−related transporter gene count
Gene count
Chemical
384
356
334
276
265
168
165
162
123
123
120
118
118
117
106
98
91
89
86
84
80
76
73
73
69
65
63
63
61
60
Tetrachlorodibenzodioxin
Acetaminophen
pirinixic acid
Ethinyl Estradiol
Cyclosporine
Carbon Tetrachloride
Benzo(a)pyrene
Estradiol
Flavonoids
Phenobarbital
7,8−Dihydro−7,8−dihydroxybenzo(a)pyrene 9,10−oxide
Diethylnitrosamine
Tamoxifen
Aflatoxin B1
Dietary Fats
Valproic Acid
palm oil
Tretinoin
arsenic trioxide
Amiodarone
Progesterone
Ozone
Selenium
Sodium Selenite
Genistein
Zidovudine
bisphenol A
Clofibric Acid
vinclozolin
Zinc
B
Gene CDS length distribution
Gene CDS length (bp)
Chemical
Total
C
Gene nonsynonymous SNP density distribution
Number of nonsynonymous SNP / CDS length
Chemical
D
Gene CNV density distribution
Number of CNV / total length
Chemical
