# Supplementary material for: Human Transporter Database: Comprehensive Knowledge and Discovery Tools in the Human Transporter Genes
Source: PLoS One. 2014 Feb 18;9(2):e88883. doi: 10.1371/journal.pone.0088883 (PMC3928311; doi:10.1371/journal.pone.0088883)
Supplement: Table S1 — Curated keywords for literature searching and corresponding category in HTD. (DOCX) [file pone.0088883.s009.docx]

### Additional file 9. Table S1. Curated keywords for literature searching and corresponding category in HTD.

| **Curated keywords** | **Category in HTD** |
| --- | --- |
| ATP-binding cassette[title] AND human[orgn] AND alive[prop] | ATP related |
| cation channel[title] AND human[orgn] AND alive[prop] | Channel |
| solute carrier family[title] AND human[orgn] AND alive[prop] | Solute Carrier Family |
| aquaporin[title] AND human[orgn] AND alive[prop] | Aquaporin |
| arsenite transporter[title] AND human[orgn] AND alive[prop] | ATP related |
| FXYD domain containing ion transport AND human[orgn] AND alive[prop] | Others |
| ATPase[title] AND human[orgn] AND alive[prop] | ATP related |
| ATP synthase[title] AND human[orgn] AND alive[prop] | ATP related |
| potassium channel[title] AND human[orgn] AND alive[prop] | Channel |
| complement component 8[title] AND human[orgn] AND alive[prop] | Defensin |
| complement component 9[title] AND human[orgn] AND alive[prop] | Defensin |
| calcium channel[title] AND human[orgn] AND alive[prop] | Channel |
| anion channel[title] AND human[orgn] AND alive[prop] | Channel |
| cholinergic receptor nicotinic[title] AND human[orgn] AND alive[prop] | Channel |
| chloride channel[title] AND human[orgn] AND alive[prop] | Channel |
| gated channel[title] AND human[orgn] AND alive[prop] | Channel |
| cytochrome c oxidase[title] AND human[orgn] AND alive[prop] | Cytochrome c oxidase |
| gamma-aminobutyric acid[title] AND receptor[title] AND human[orgn] AND alive[prop] | Channel |
| GABA-A receptor activity[go] AND human[orgn] AND alive[prop] | Channel |
| gap junction protein[title] AND human[orgn] AND alive[prop] | Gap junction |
| connexin[title] AND human[orgn] AND alive[prop] | Gap junction |
| glycine receptor[title] AND human[orgn] AND alive[prop] | Channel |
| glutamate receptor ionotropic[title] AND human[orgn] AND alive[prop] | Channel |
| inositol triphosphate receptor AND human[orgn] AND alive[prop] | Channel |
| ion channel[title] AND human[orgn] AND alive[prop] | Channel |
| transmembrane transport[go] AND human[orgn] AND alive[prop] |  |
| ion transmembrane transporter activity[go] AND human[orgn] AND alive[prop] |  |
| Rh blood group[All Fields] AND human[orgn] AND alive[prop] | Others |
| ryanodine receptor[title] AND human[orgn] AND alive[prop] | Channel |
| sodium channel[title] AND human[orgn] AND alive[prop] | Channel |
| uncoupling protein[title] AND human[orgn] AND alive[prop] | Solute Carrier Family |
| bestrophin[title] AND human[orgn] AND alive[prop] | Channel |
| anoctamin[title] AND human[orgn] AND alive[prop] | Channel |
| synaptic vesicle glycoprotein[title] AND human[orgn] AND alive[prop] | Others |
| major facilitator superfamily[title] AND human[orgn] AND alive[prop] | Others |
| polycystic kidney disease[title] AND human[orgn] AND alive[prop] | Channel |
| pannexin[title] AND human[orgn] AND alive[prop] | Gap junction |
| six transmembrane epithelial antigen of the prostate AND human[orgn] AND alive[prop] | Channel |
| plasmolipin[title] AND human[orgn] AND alive[prop] | Channel |
| Rh family[title] AND human[orgn] AND alive[prop] | Others |
| two pore segment channel AND human[orgn] AND alive[prop] | Channel |
| mucolipin AND human[orgn] AND alive[prop] | Channel |
| SV2 related protein homolog AND human[orgn] AND alive[prop] | Others |
| tweety homolog AND human[orgn] AND alive[prop] | Channel |
| sideroflexin AND human[orgn] AND alive[prop] | Others |
| magnesium transporter[title] AND human[orgn] AND alive[prop] | Others |
| mitochondrial carrier triple repeat AND human[orgn] AND alive[prop] | Solute Carrier Family |
| organic solute transporter[title] AND human[orgn] AND alive[prop] | Others |
| defensin[title] AND human[orgn] AND alive[prop] | Defensin |
| nucleoporin[title] AND human[orgn] AND alive[prop] | Nucleoporin |
| transmembrane channel[title] AND human[orgn] AND alive[prop] | Channel |
| chloride channel[All Fields] AND human[orgn] AND alive[prop] | Channel |
| sodium channel[All Fields] AND human[orgn] AND alive[prop] | Channel |
| potassium channel[All Fields] AND human[orgn] AND alive[prop] | Channel |
