# Supplementary material for: Human Transporter Database: Comprehensive Knowledge and Discovery Tools in the Human Transporter Genes
Source: PLoS One. 2014 Feb 18;9(2):e88883. doi: 10.1371/journal.pone.0088883 (PMC3928311; doi:10.1371/journal.pone.0088883)
Supplement: Table S2 — Annotation entry statistics for 1555 human transporter genes. (DOC) [file pone.0088883.s010.doc]

### Additional File 10. Table S2.Annotation entry statistics for 1555 human transporter genes.

| **Data Source** | **Related entries** | **Annotated genes** | **Percent of annotated genes (%)** | **Reference** |
| --- | --- | --- | --- | --- |
| **Basic information** | | | |  |
| NCBI-Gene | 1505 | 1505 | 96.8 | (25) |
| UniProt | 1273 | 1143 | 73.5 | (26) |
| NCBI-HomoloGene | 2092 | 1079 | 69.4 | (28) |
| **Function** | | | |  |
| GO | 5761 | 1107 | 71.2 | (51) |
| InterPro (domain) | 6978 | 1077 | 69.3 | (27) |
| HPRD (PPI) | 2320 | 486 | 31.3 | (32) |
| **Expression** | | | |  |
| NCBI-UniGene (EST) | 77475 | 1029 | 66.2 | (25) |
| Allen Brain Atlas | 2941 | 172 | 11.1 | (33) |
| RNA-seq from (8) | 1028 | 1025 | 65.9 | (34) |
| RNA-seq from (9) | 255 | 255 | 16.4 | (35) |
| **Genomic Variation** | | | |  |
| dbSNP & HapMap (SNP) | 1197016 | 1279 | 82.3 | (29,30) |
| DGV (CNV) | 4040 | 855 | 55.0 | (31) |
| **Epigenetics & Regulation** TF binding sites | 156102 | 1295 | 83.3 | (36) |
| **Pathway, Disease, Drug/Chemical** | | | |  |
| KOBAS (pathway) | 2566 | 652 | 41.9 | (40) |
| KOBAS (disease) | 3083 | 530 | 34.1 | (40) |
| PharmGKB | 3014 | 257 | 16.5 | (37) |
| CTD | 141370 | 1043 | 67.1 | (38) |
| DrugBank | 2732 | 273 | 17.6 | (39) |
| Transporter substrate | 1417 | 701 | 45.1 | (17) |

**REFERENCE**

1. Zhao, M., Chen, Y., Qu, D. and Qu, H. (2011) TSdb: a database of transporter substrates linking metabolic pathways and transporter systems on a genome scale via their shared substrates. *Sci China Life Sci*, **54**, 60-64.
2. Sayers, E.W., Barrett, T., Benson, D.A., Bolton, E., Bryant, S.H., Canese, K., Chetvernin, V., Church, D.M., DiCuccio, M., Federhen, S. et al. (2011) Database resources of the National Center for Biotechnology Information. *Nucleic Acids Res*, 39, D38-51.
3. UniProtConsortium. (2010) The Universal Protein Resource (UniProt) in 2010. *Nucleic Acids Res*, 38, D142-148.
4. Burge, S., Kelly, E., Lonsdale, D., Mutowo-Muellenet, P., McAnulla, C., Mitchell, A., Sangrador-Vegas, A., Yong, S.Y., Mulder, N. and Hunter, S. (2012) Manual GO annotation of predictive protein signatures: the InterPro approach to GO curation. *Database* (Oxford), 2012, bar068.
5. Sayers, E.W., Barrett, T., Benson, D.A., Bolton, E., Bryant, S.H., Canese, K., Chetvernin, V., Church, D.M., Dicuccio, M., Federhen, S. et al. (2012) Database resources of the National Center for Biotechnology Information. *Nucleic Acids Res*, 40, D13-25.
6. Sherry ST, Ward MH, Kholodov M, Baker J, Phan L, et al. (2001) dbSNP: the NCBI database of genetic variation. Nucleic Acids Res 29: 308-311.
7. International HapMap Consortium. (2010) Integrating common and rare genetic variation in diverse human populations. Nature 467(7311):52-58.
8. Zhang, J., Feuk, L., Duggan, G.E., Khaja, R. and Scherer, S.W. (2006) Development of bioinformatics resources for display and analysis of copy number and other structural variants in the human genome. *Cytogenet Genome Res*, 115, 205-214.
9. Keshava Prasad, T.S., Goel, R., Kandasamy, K., Keerthikumar, S., Kumar, S., Mathivanan, S., Telikicherla, D., Raju, R., Shafreen, B., Venugopal, A. et al. (2009) Human Protein Reference Database--2009 update. Nucleic Acids Res, 37, D767-772.
10. Jones, A.R., Overly, C.C. and Sunkin, S.M. (2009) The Allen Brain Atlas: 5 years and beyond. Nat Rev Neurosci, 10, 821-828.
11. Wang, E.T., Sandberg, R., Luo, S., Khrebtukova, I., Zhang, L., Mayr, C., Kingsmore, S.F., Schroth, G.P. and Burge, C.B. (2008) Alternative isoform regulation in human tissue transcriptomes. Nature, 456, 470-476.
12. Wu, J.Q., Habegger, L., Noisa, P., Szekely, A., Qiu, C., Hutchison, S., Raha, D., Egholm, M., Lin, H., Weissman, S. et al. (2010) Dynamic transcriptomes during neural differentiation of human embryonic stem cells revealed by short, long, and paired-end sequencing. Proc Natl Acad Sci U S A, 107, 5254-5259.
13. Karolchik D, Barber GP, Casper J, Clawson H, Cline MS, et al. (2014) The UCSC Genome Browser database: 2014 update. Nucleic Acids Res 42: D764-770.
14. Relling, M.V., Gardner, E.E., Sandborn, W.J., Schmiegelow, K., Pui, C.H., Yee, S.W., Stein, C.M., Carrillo, M., Evans, W.E. and Klein, T.E. (2011) Clinical Pharmacogenetics Implementation Consortium guidelines for thiopurine methyltransferase genotype and thiopurine dosing. Clin Pharmacol Ther, 89, 387-391.
15. Davis, A.P., King, B.L., Mockus, S., Murphy, C.G., Saraceni-Richards, C., Rosenstein, M., Wiegers, T. and Mattingly, C.J. (2011) The Comparative Toxicogenomics Database: update 2011. Nucleic Acids Res, 39, D1067-1072.
16. Knox, C., Law, V., Jewison, T., Liu, P., Ly, S., Frolkis, A., Pon, A., Banco, K., Mak, C., Neveu, V. et al. (2011) DrugBank 3.0: a comprehensive resource for 'omics' research on drugs. Nucleic Acids Res, 39, D1035-1041.
17. Xie, C., Mao, X., Huang, J., Ding, Y., Wu, J., Dong, S., Kong, L., Gao, G., Li, C.Y. and Wei, L. (2011) KOBAS 2.0: a web server for annotation and identification of enriched pathways and diseases. Nucleic Acids Res, 39, W316-322.
18. Ashburner,M., Ball,C.A., Blake,J.A., Botstein,D., Butler,H.,Cherry,J.M., Davis,A.P., Dolinski,K., Dwight,S.S., Eppig,J.T. et al.(2000) Gene ontology: tool for the unification of biology. The GeneOntology Consortium. *Nat. Genet.*, 25, 25–29.
